# Photoresponse of new azo pyridine functionalized poly(2-hydroxyethyl methacrylate-*co*-methyl methacrylate)

**DOI:** 10.1038/s41598-024-59704-1

**Published:** 2024-04-20

**Authors:** Jolanta Konieczkowska, Dorota Neugebauer, Anna Kozanecka-Szmigiel, Aleksy Mazur, Sonia Kotowicz, Ewa Schab-Balcerzak

**Affiliations:** 1 Centre of Polymer and Carbon Materials, Polish Academy of Sciences, 34 M. Curie-Sklodowska Str., 41-819 Zabrze, Poland; 2 Department of Physical Chemistry and Technology of Polymers, Faculty of Chemistry, Silesian University of Technology, Ks. Marcina Strzody 9, 44-100 Gliwice, Poland; 3 Faculty of Physics, Warsaw University of Technology, 75 Koszykowa Str., 00-662 Warsaw, Poland; 4 Institute of Chemistry, University of Silesia, 9 Szkolna Str., 40-006 Katowice, Poland

**Keywords:** Optical materials, Soft materials, Optical physics, Polymers, Polymer synthesis

## Abstract

A new azo polymer containing photoisomerizable azo pyridine functionalities was synthesized via Mitsunobu reaction of 4-(4-hydroxyphenylazo)pyridine with poly(2-hydroxyethyl methacrylate-*co*-methyl methacrylate) (p(HEMA-*co*-MMA)) for creating new photochromic materials. The resulting polymer with azo pyridine side groups was characterized for structural, thermal, and optical properties. UV–vis, ^1^H NMR and IR spectroscopies confirmed that all hydroxyl groups in p(HEMA-*co*-MMA) were substituted with azo dye. The obtained azo copolymer exhibited high thermal stability (around 240 °C) and a glass transition temperature (113 °C), promising for applications. The *trans*-to-*cis* isomerization upon UV irradiation and the thermal back reaction of the azo chromophore in the copolymer in the solid state was studied. A photostationary state with 50% content of *cis*-isomers upon 6 min of UV irradiation was reached, and during 48 h dark relaxation at ambient temperature, all *cis*-isomers converted to the *trans* form. Additionally, the possibility of efficient photogeneration of surface relief gratings with high amplitude of azo copolymer surface modulation was demonstrated.

## Introduction

Light-responsive materials play a key role in modern optoelectronic, photonic, biological, and biomedical applications^1^. Among various photoresponsive systems, azobenzene derivatives also known as azoarenes or azo dyes, due to their ability for reversible one or two-photon-induced isomerization still hold a prominent position and offer- a wide range of functionalities^2,3^. Thermodynamically stable *trans* (E) isomer under irradiation with UV–vis radiation forms metastable *cis* (Z) isomer, which can be converted back to the E isomer via thermal or light-induced reaction. Beyond typical applications of azo compounds being colored like dyes in the chemical industry are attractive for many modern directions of utilization. During the past decades, photoresponsive systems based on azobenzene were investigated for photonics, for information storage^4^, as photoaligning layer for liquid crystals^5^ as photomechanical response materials^6,7^. However, the possibility of azo materials application is still extended. Recently, azobenzene derivatives have been investigated as suitable compounds for molecular solar thermal storage (MOST), which is a unique strategy for solar thermal energy conversion and storage^3^. It was found that adding azobenzene to the perovskite layer improved the optical, thermal, and structural stability of the photovoltaic perovskite solar cells^8^. Azobenzenes are interesting compounds for biomedical applications^1^. They have been utilized for the preparation of biomaterials, which are particularly helpful in elucidating cells that adapt to a dynamic microenvironment or integrate spatiotemporal variations of signals^9^. Azo polymers have been also applied for the synthesis of amphiphilic block copolymers for the preparation of light-responsive micelles as a potential drug delivery system for melanoma^10^. Triblock polymer brushes in which one block contains azobenzene units have been used for the fabrication of smart nonwoven fabric showing great potential in treating compilated polluted water from most industrial fields^11^. Thus, there is still a need for designing and preparation of novel azo materials with enhanced functionalities via the synthesis of new azobenzene derivatives or their incorporation into polymers or other materials, which enable isomerization in the solid state giving the opportunity for the construction of innovative materials^12–14^. Various types of azo polymers have been developed. The architecture of azo polymers plays an important role in their photoresponsive properties, functions, and applications. Considering the chemical structure of polymer backbone forming azo system, they can be divided into three groups, that is, aliphatic (for example based on PMMA^15^ or based on PS-b-PMMA^16^), aliphatic–aromatic and aromatic (for example azo polyimides^17–19^) azo polymers. Recently, new aliphatic azobenzene, both functionalized and supramolecular (co)polymers have been developed^20,21^.

In our investigations presented in this work, we focused on the synthesis of new azo polymers, in which the polymer backbone is formed by poly(2-hydroxyethyl methacrylate-*co*-methyl methacrylate) (p(HEMA-*co*-MMA)). Generally, poly(2-hydroxyethyl methacrylate) (pHEMA) is optically transparent, biocompatible, cytocompatible, non-degradable, hydrophilic methacrylate-based polymer^22^. It is considered a non-toxic biomaterial with excellent biocompatibility, which can absorb and retain water within its structure, forming an oxygen-permeable hydrogel, characterized by good chemical stability, flexibility, and high water content making it suitable for various biomedical applications^23^. pHEMA has found application in different electronic devices. In the case of stretchable electronics, the hydrogel-elastomer-based soft electronic circuits have been produced^24^. Regarding optoelectronics devices, pHEMA-TiO_2_ hybrid demonstrated efficient light-induced separation of charges^25,26^. Nanocomposites based on pHEMA hydrogels and conductive material, due to the piezoelectric characteristics, are also candidate materials for self-powered and energy harvesting devices, which work in aqueous conditions^27^. HEMA can be either polymerized or copolymerized to modify the properties of the resulting polymer. Furthermore, the available hydroxyl groups in the (co)polymer chain offer opportunity for the functionalization reactions including protection group or esterification, which leads to the preparation of activated bromides, so-called multifunctional macroinitiators used in ATRP (atom transfer radical polymerization)^28–30^. Therefore, advanced functional copolymers, e.g. drug delivery carriers, with various topologies ranging from linear, star-shape to grafted, have been synthesized^29–33^. The presence of hydroxyl groups in the p(HEMA-*co*-MMA) also gives an opportunity for the introduction of the azo compound via the Mitsunobu reaction, which has already been used to modify polyesterimides^17^. Such a reaction so far has not been applied for the covalent bonding of azo chromophore with pHEMA. Azobenzene-functionalized copolymers based on HEMA have been synthesized by an esterification reaction after the conversion of the azo compound to azobenzene acid chloride^34–36^. Thus, utilization of the Mitsunobu reaction allows for simplifying the azo functionalization of pHEMA because the phenol group in azo chromophore may react directly with pHEMA through –OH species.

Herein, azo polymer with covalently bonded azo dye was obtained by functionalization of synthesized p(HEMA-*co*-MMA) with 4-(4-hydroxyphenylazo)pyridine (1:1). Considering the literature review there are no articles that described azo polymers based on pHEMA and azo pyridine derivatives. Based on our previous works it can be concluded, that azo pyridines show significantly faster *cis–trans* isomerization in dark^37^, and can improve the thermal properties of polymers in comparison to azobenzene analogues^38^.

The prepared azo(HEMA-*co*-MMA) was characterized considering its ability for photoisomerization in a solid state and the possibility for creating photonic structures, that is, surface relief gratings (SRGs). The results from the carried out investigations prove the application potential of the new azo polymer.

## Results and discussion

**Figure 1:**
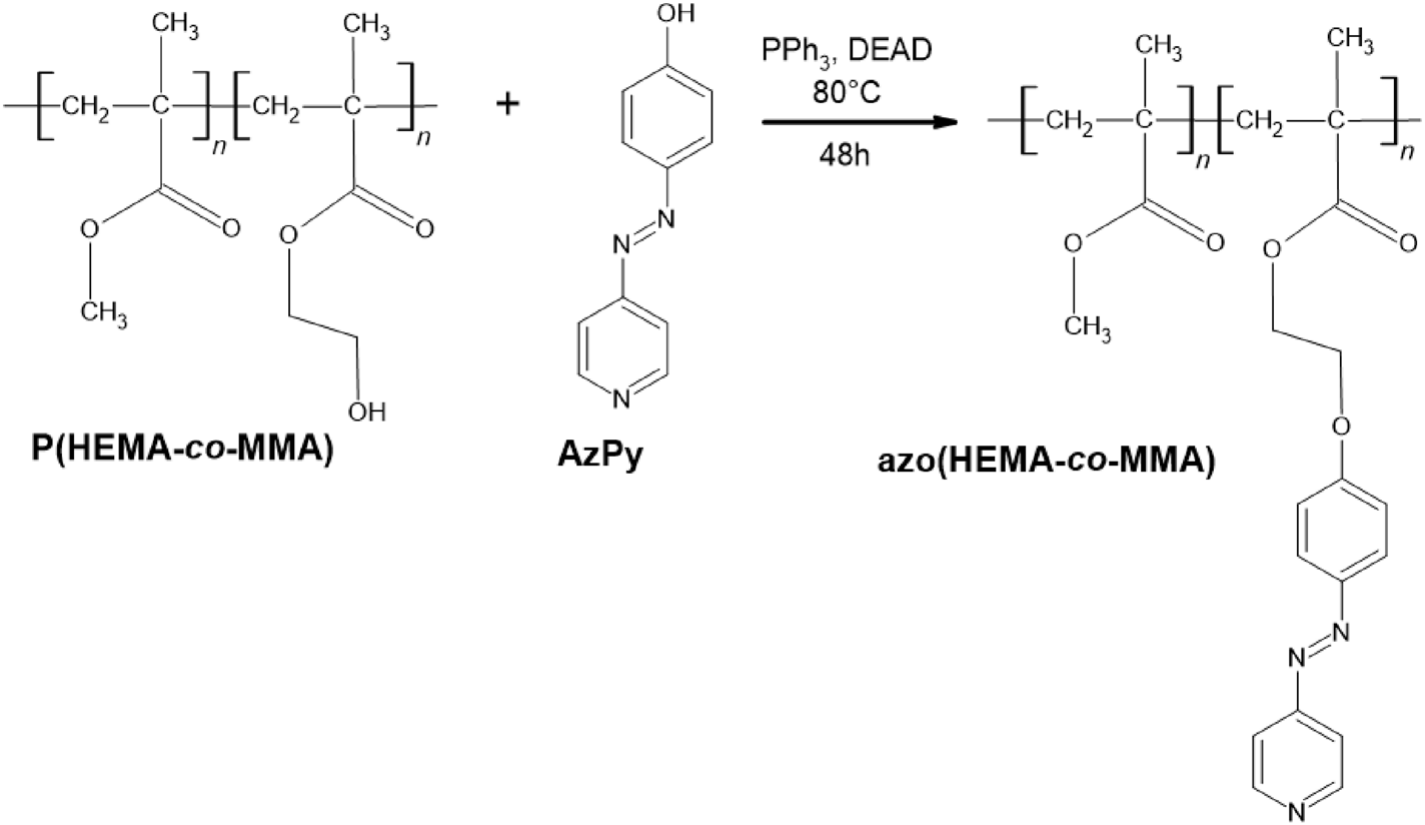
Chemical synthesis route of **azo(HEMA-*co*-MMA)**.

New azo pyridine-modified poly(2-hydroxyethyl methacrylate-*co*-methyl methacrylate) denoted as **azo(HEMA-*co*-MMA)** was synthesized and characterized considering its thermal, UV–vis absorption properties, *cis–trans* isomerization process and the ability for surface relief grating photogeneration. In the first step, poly(2-hydroxyethyl methacrylate-*co*-methyl methacrylate) denoted as **p(HEMA-*co*-MMA)** was synthesized via atom transfer radical polymerization (ATRP) of 2-hydroxyethyl methacrylate (HEMA) and methyl methacrylate (MMA) using the initial ratio of 50/50. The resulting MMA/HEMA content (48/52) in the copolymer was determined by comparison of the integrals of the peaks in the ^1^H NMR spectrum (details in *Supporting Information*). GPC analysis was applied to estimate the revealed average molar mass of **p(HEMA-*co*-MMA)**. It should be stressed that the obtained values of molecular masses should be treated only indicatively. Absolute molar masses may differ from the calculated based on the calibration of hydrodynamic values of the studied polymers differ from those of the polystyrene standards. The revealed number (M_n_) was 16 900 g/mol with a relatively low dispersity (1.41). The presence of a pendant aliphatic hydroxyl group allows further functionalization. Functionalized **azo(HEMA-*co*-MMA)** was obtained by the Mitsunobu reaction between **p(HEMA-*co*-MMA)** and 4-(4-hydroxyphenylazo)pyridine (**AzPy**). Details of the chemical synthesis of **p(HEMA-*co*-MMA)** and **azo(HEMA-*co*-MMA)** (Fig. 1) are presented in *Supporting Information* in Sect. 1.2 and 1.3.

The substitution of hydroxyl groups in the **p(HEMA-*co*-MMA)** precursor was determined by the UV–Vis spectroscopy using the Lambert–Beer Law^18^. The degree of chromophore substitution in the polymer was calculated from the calibration curves obtained from the UV–Vis spectra of the **AzPy** solutions with various concentrations (Fig. S1 in *support. inf.*). From the comparison of the peak absorbance of the polymer in NMP solution with the known concentration and the calibration curve, the degree of the functionalization was estimated as 100%. The total substitution of **p(HEMA-*co*-MMA)** hydroxyl groups with **AzPy** was also confirmed by FTIR and ^1^H NMR spectroscopies. ^1^H NMR spectrum of **p(HEMA-*co*-MMA)** precursor showed the hydroxyl proton peak at 4.87 ppm, which disappeared after the Mitsunobu reaction. At the same time, new signals appeared in the range of 7.00–8.67 ppm characteristic of aromatic protons of azo chromophore. The FTIR spectra of **p(HEMA-*co*-MMA)** showed absorption bands in the range of 3100–3600 cm^-1^ attributed to hydroxyl groups. Absorption bands in the range of 2800–3000 cm^-1^ are characteristic of C–H bonds. The presence of C=O and C–O bonds was observed as absorption bands at ca. 1750 cm^-1^ and 1150 cm^-1^, respectively. After **AzPy** functionalization new absorption bands appeared at 1587 cm^-1^ and 988 cm^−1^ attributed to the N=N group and pyridine ring, respectively.

Wide-angle X-ray diffraction measurements performed for **azo(HEMA-*co*-MMA)** showed a diffraction pattern with one broad peak in the range of 10–35° of the diffusion type typical for amorphous materials (Fig. S2). It should be noticed that despite the high degree of azo pyridine substitution no signals characteristic for crystalline domains was observed.

The thermal properties of synthesized polymers were evaluated by thermogravimetric analysis (TGA) and differential scanning calorimetry (DSC) in a nitrogen atmosphere. The data are collected in Table 1. **P(HEMA-*co*-MMA)** precursor exhibited one thermal degradation step with the temperature of the maximum decomposition rate (T_max_) at 395 °C (Fig. S3 in *support. inf.*). The incorporation of azo pyridine units resulted in appearing of a new degradation step at 272 °C, characteristic of the thermal decomposition of **AzPy**. The 5% and 10% weight loss temperature (T_5_, T_10_) are usually considered as the criterion determining the thermal stability of polymers. Synthesized polymers exhibited high thermal stability above 240 °C (Table 1). The incorporation of azo moieties into the polymer structure resulted in decreased thermal stability in comparison to the **p(HEMA-*co*-MMA)**. The glass transition temperature (*T*_*g*_) of **p(HEMA-*co*-MMA)** was 97 °C. After azo pyridine functionalization, *T*_*g*_ increased to 113 °C (Fig. 4 in *support. inf.*). In general, the incorporation of azo groups into the polymer structure results in a decrease in the *T*_*g*_ due to the plasticization effect of the chromophore. In the case studied **azo(HEMA-*co*-MMA)** the increase of the *T*_*g*_ can be the result of the high content of azo chromophore and its high *T*_*g*_ (263 °C).

**Table 1:** DSC and TGA data for **p(HEMA-*co*-MMA)**, **azo(HEMA-*co*-MMA)**, and azo pyridine.

Polymer code	DSC		TGA[N_2_]	
	T_g_ [°C]	T_5_ [°C]^a^	T_10_ [°C]^a^	T_max_ [°C]^b^
p(HEMA-*co*-MMA)	97	279	329	395
azo(HEMA-*co*-MMA)	113	246	264	272; 427
AzPy^[54]^	263	240	250	277

^a^T_5_/T_10_—temperature based on 5%/10% weight loss obtained from TGA curves.

^b^T_max_—Temperature of the maximum decomposition rate from DTG curves.

TGA was measured in the range of 25–800 °C.

### *Cis–trans* isomerization

**Figure 2:**
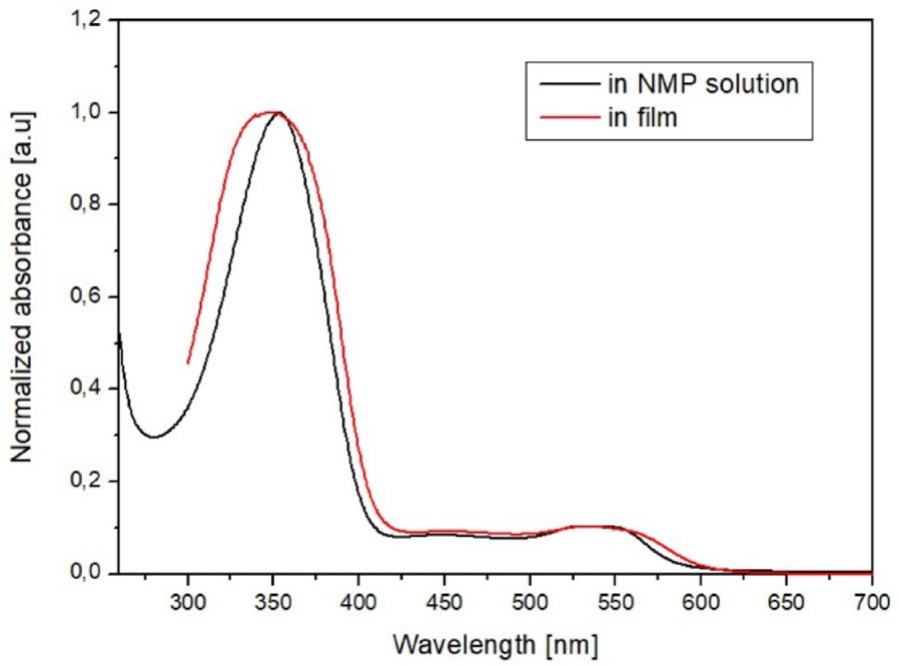
Normalized UV–Vis curves for **azo(HEMA-*co*-MMA)** in NMP solution (c = 10^–5^ mol/l) and the solid state. Thickness of the film was ca. 300 nm.

Optical properties of **azo(HEMA-*co*-MMA)** were investigated both in NMP solution (c = 10^–5^ mol/l) and in the solid state as films cast on a glass substrate. Preparation of the film and isomerization measurements details using UV–Vis spectroscopy is described in *Support. Inf.* (Sec. 1.4 and 2.3). The thickness of the **azo(HEMA-*co*-MMA)** film was ca. 300 nm. The UV–Vis spectra are shown in Fig. 2.

Azo polymer **azo(HEMA-co-MMA)** exhibited broad absorption within the range of 260–600 nm, featuring a prominent absorption band with a maximum (λ_max_) situated at approximately 349 nm, a characteristic trait of azo chromophores. The absorption spectra of the polymer film appeared broader and marginally blue-shifted in contrast to the solution (Fig. 2). It may suggest the occurrence of H-aggregation among chromophores in the solid-state^39^.

**Figure 3:**
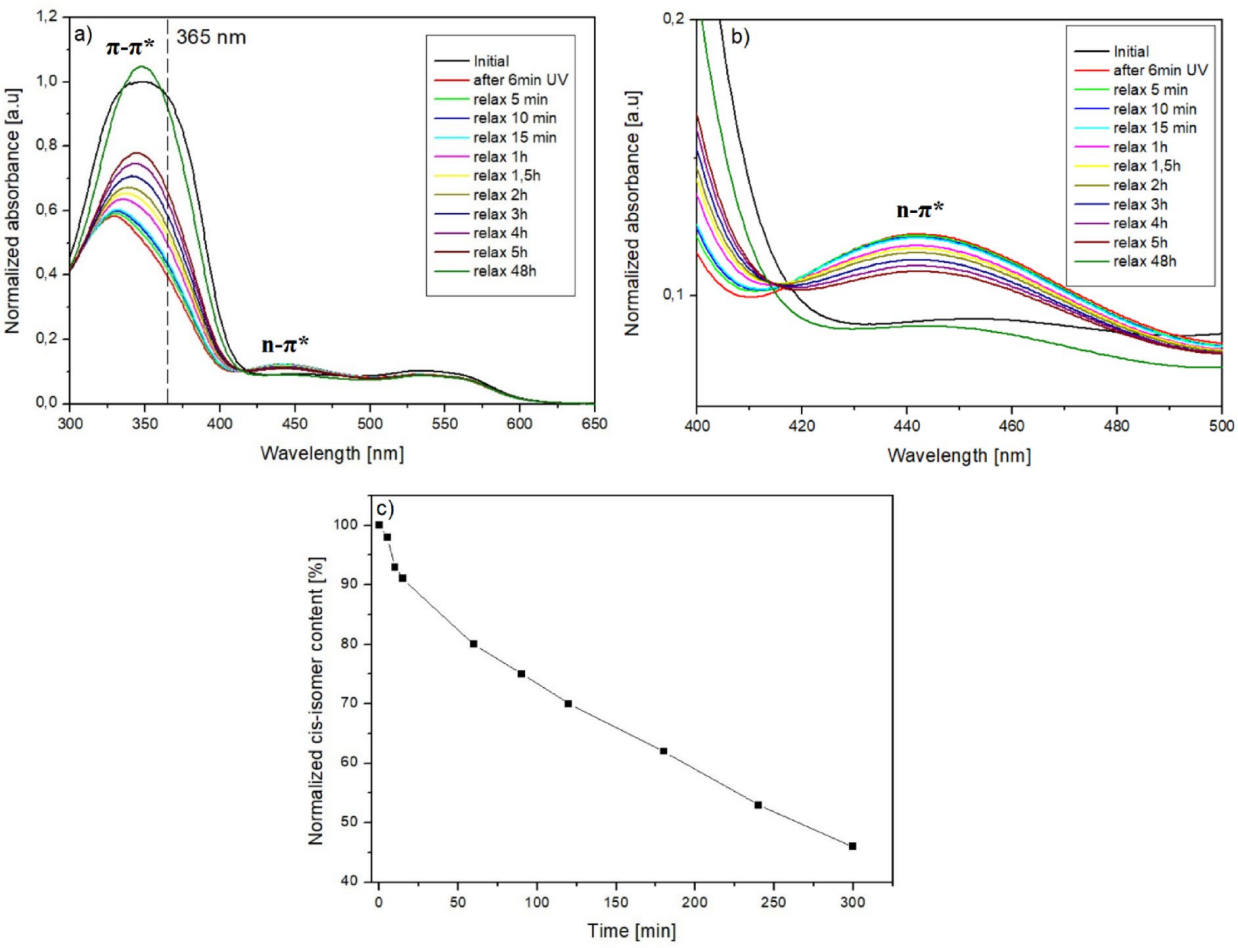
Curves of *cis–trans* isomerization for **azo(HEMA-*co*-MMA)** (**a**) whole range of spectra; (**b**) in the range of absorption characteristic for π-π*. The 365 nm excitation radiation was marked as a dashed line. (**c**) Normalized content of *cis*-isomer in the function of time.

The dark *cis–trans* isomerization was investigated for **azo(HEMA-*co*-MMA)** in the solid state. Non-irradiated **azo(HEMA-*co*-MMA)** film exhibited an absorption area in the range of 300–600 nm. High-intensity band located at 349 nm is attributed to the π–π* transition of the *trans*-isomer, corresponding to the 100% of the *trans*-isomer content in the sample. A weak band in the range of 425–600 nm is attributed to the n–π* transition of *trans*-isomer (black curve in Fig. 3a,b). The polymer film was irradiated with 365 nm light located near the maximum of the azo chromophore absorption. Exposure to excitation radiation resulted in a reduction in absorption band intensity at λ_max_ 349 nm and a shift towards a shorter wavelength. At the same time, an increase in the intensity of the band in the range of 425–475 nm and the formation of the isosbestic points at 417 and 492 nm were observed (red curve in Fig. 3a,b).

The *trans* to *cis*-isomer photoconversion efficiency (%) was determined by monitoring the change in the absorbance at the wavelength corresponding to the absorption maximum of the *trans*-form, before and immediately after light irradiation. *Cis*-isomer content was calculated using the following equation^40^:

1 $$P=\frac{{A}_{0}-{A}_{t}}{{A}_{0}} \times 100\%$$

where *A*_*0*_ and *A*_*t*_ correspond to the maximum normalized absorbance, respectively before and after reaching the photostationary state. After 6 min of UV-light irradiation the **azo(HEMA-*co*-MMA)** reached the photostationary state where 50% of *cis*-isomers were generated.

After turning off the excitation beam, **azo(HEMA-*co*-MMA)** returned to its initial state as a result of the *cis*–*trans* back reaction. Directly after switching off the excitation light, the intensity of the n–π* band was reduced, while the π–π* band was increased (Fig. 3a,b). Changes in the absorption spectra were monitored during 48 h. After total dark relaxation, the UV–Vis spectra reached the initial state (dark green curve in Fig. 3a,b) with different characteristics than the spectra measured before UV light exposure (black curve in Fig. 3a,b). The curve was higher and taller than the curve measured before excitation light irradiation. The same difference in the character of UV–Vis spectra is observed for curves measured in NMP solution and film.

(Fig. 2). It may result from different orientations and stacking of azo pyridine groups in the sample before and after irradiation^41^. After UV-light irradiation, some of these interactions are broken; as a result the spectrum is similar to this obtained for the NMP solution (Fig. S6 in *support. inf.*). Figure 3c presents the dynamic of *cis–trans* isomerization as a decrease of the normalized content of *cis*-isomer in the function of time. **Azo(HEMA-*co*-MMA)** showed relatively slow *cis–trans* relaxation, where almost 55% of generated *cis*-isomers converted to the *trans* form after 300 min of turning off the excitation light. The total conversion to the *trans*-isomer was observed after 48 h. The dynamic of the *cis–trans* reaction of **azo(HEMA-*co*-MMA)** is difficult to compare to literature results. There are no investigations of dark *cis–trans* relaxation **azo(HEMA-*co*-MMA)**. In paper^42^ PMMA doped derivative of azobenzene derived from the amino acid, *cis–trans* reaction was monitored at room temperature, where ca. 50% of *cis*-isomer converted to *trans* form within 4 h after turning off the excitation light. For PMMA-doped heterocyclic azo dyes, the dark relaxation is strongly dependent on the content of *cis*-isomer. For rich *cis*-isomer polymer, ca. 50% of *cis*-isomer converted to the *trans* form within 60 min^43^.

### Surface relief gratings

Details regarding the applied holographic grating recording and monitoring experimental conditions are described in *Supporting Information* (Section 2.4). According to the results presented in Sect. “Cis–trans isomerization” the excitation wavelength used in studying the process of surface relief grating formation (i.e., 457 nm) fell at the n–π* absorption bands of both the *trans* and *cis* isomers in **azo(HEMA-*co*-MMA)**. Such irradiation conditions were expected to ensure efficient *cis–trans* photoisomerization cycling. The probe wavelength (690 nm) was outside the **azo(HEMA-*co*-MMA)** absorption band.

**Figure 4:**
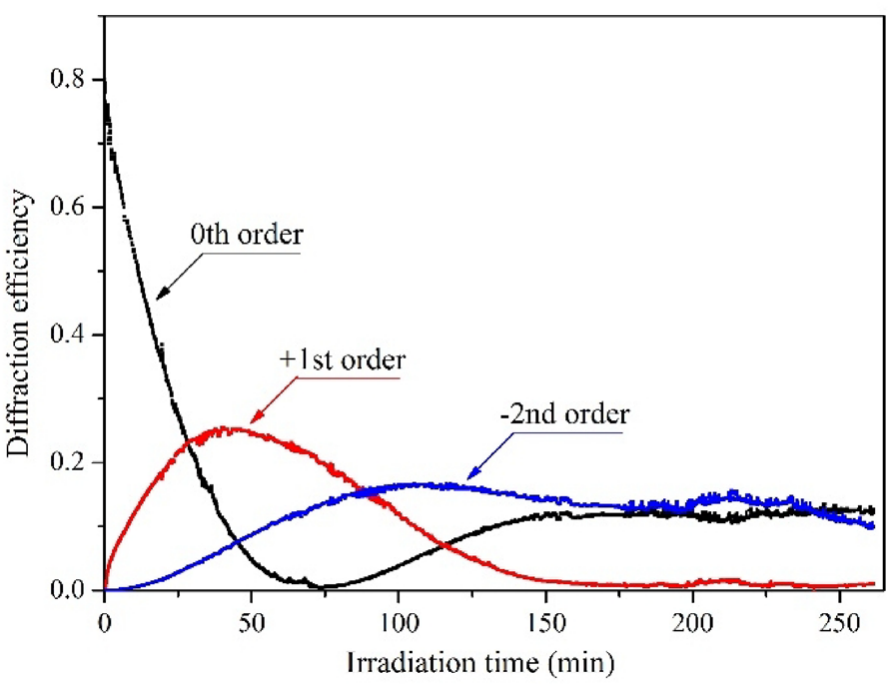
Evolution of the 0th, + 1st, -2nd order diffraction efficiencies of the SRG inscribed in the **azo(HEMA-*co*-MMA)** layer during irradiation with a light polarization pattern.

Figure 4 shows the detected plots of the temporal evolution of the 0th, +1st, and −2nd order diffraction efficiency during over 4-h SRG inscription process (the diffraction efficiency of a particular order was calculated as a fraction of incident optical power diffracted into that diffraction order). Switching on the 457 nm irradiation resulted in an immediate decrease in the optical power of the non-diffracted light, which was accompanied by an increase in the optical power of the beams diffracted into the first orders; the second-order beams appeared slightly later. After 45 min from the beginning of the exposure, the first-order diffraction signals reached their maximum values of 25%, and within the next 25 min, the zeroth order beam was practically diminished. Further irradiation led to extinguishing the first diffraction orders at t = 180 min. Interestingly, no other essential changes in the plots of diffracted signals were observed until turning off the 457 nm beams.

**Figure 5:**
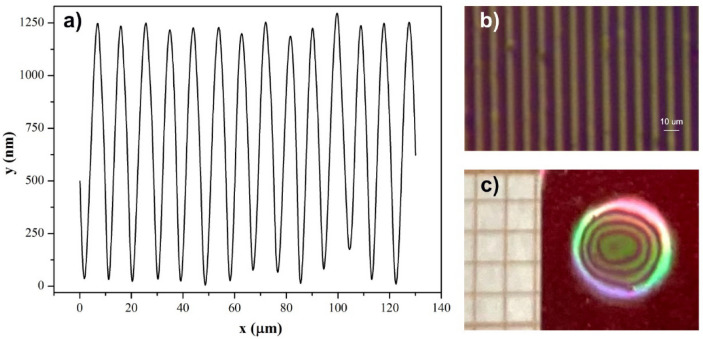
(**a**) The topography profile of the **azo(HEMA-co-MMA)** surface measured after sample irradiation with a light polarization pattern. (**b**) The optical microscopy image referring to the center of the generated SRG. (**c**) The photograph of the sample surface with the inscribed SRG observed under white light; a piece of graph paper on the left provides the scale.

All measured diffraction efficiency curves evolved with time showing general trends expected for the sinusoidal phase gratings, i.e., the squared Bessel functions of the zeroth, first and second order^44^. This observation is similar to what we found recently for holographically generated SRG in functionalized azo poly(ether imide)^45^. Detecting the extinguished 0th and 1st diffraction order signals indicated an inscription of a deep grating in **azo(HEMA-co-MMA)**. Indeed, the sample profile measured after irradiation revealed a relief structure with the maximum peak-to-valley height reaching a value as large as 1200 nm (Fig. 5a). The period of the surface modulation was ca. 9.3 µm, thus agreeing with the spatial period of the light interference pattern and proving the light-induced origin of the relief structure. The lack of essential changes in the diffraction efficiency signals found during the last hour of irradiation suggests that 1200 nm is the maximum SRG depth achievable in **azo(HEMA-co-MMA)** under the applied irradiation conditions. The result might also indicate an existence of a limit in the SRG formation process in azo polymers in general.

Figure 5b presents the optical microscopy image, referring to the centre of the generated SRG. The expected regular pattern showing a one-dimensional grating structure was observed. Interestingly, as demonstrated in Fig. 5c, the effect of the inscription of a deep SRG on the polymer surface was easily seen by the naked eye. When tilting the sample, the circular structure with dark rings was seen together with a colour rainbow. The structure size was consistent with the size of circular spots of the blue interfering beams (*support.inf.* Section 2.4).

**Figure 6:**
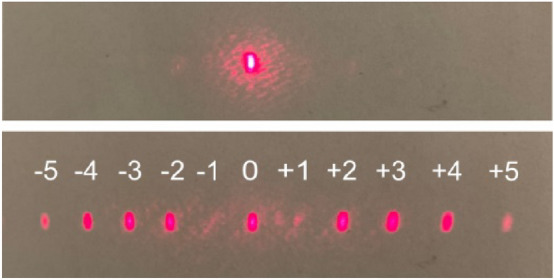
Photograph of diffraction pattern (bottom row) observed on the screen behind the **azo(HEMA-*co*-MMA)** when the 690 nm beam was incident on the 1200 nm-deep SRG compared to the photograph of the non-diffracted 690 nm beam spot (top row) observed on the screen when the probe beam was incident on the sample outside the SRG area.

Figure 6 (the bottom row) shows the photograph of the diffraction pattern observed on the screen behind the **azo(HEMA-*co*-MMA)** sample when the 690 nm probe beam was incident normally on the centre of the holographically irradiated area (after turning off the writing beams). As can be seen, the 1200 nm-deep structure generated multiple diffraction orders up to the 5th ones and allowed for complete suppression of the first diffraction orders.

Considering the literature results concerning azo polymers with aliphatic backbone it was found that scientific interest is mainly focused on commercially available poly[1-[4-(3-carboxy-4 hydroxyphenylazo)benzenesulfonamido]-1,2-ethanediyl, sodium salt (PAZO). In PAZO, which has been modified by Au or TiO_2_ or Ag or Ag/Au nanoparticles to obtain high surface modulation, the SRGs with amplitude from 130 to 360 nm and about 337–500 nm have been reached during 10 h of irradiation depending on nanoparticle type, its size, amount and recording angle 2θ^46–48^. The highest modulation depth of about 950 nm was obtained in PAZO during 14 h of irradiation^49^.

## Conclusions

A new azo pyridine functionalized poly(2-hydroxyethyl methacrylate-*co*-methyl methacrylate) was obtained in two-step synthesis involving ATRP copolymerization of MMA and HEMA and substitution of hydroxyl units with 4-(4-hydroxyphenylazo)pyridine. It is the first example of an azo pyridine-functionalized methacrylate copolymer. **Azo(HEMA-co-MMA)** showed photoisomerization when exposed to UV light (356 nm) and relatively fast thermal back reaction in the film compare to polymers with aromatic backbone. We demonstrated a very efficient light-induced process of SRG inscription in **azo(HEMA-*co*-MMA)**. The modulation depth of the ca. 9 µm-period structure reached 1200 nm, which is among the largest values reported so far for SRGs formed holographically in methacrylate-based azo polymers. Simultaneously, the detected curves of the time evolution of diffraction efficiency during holographic grating recording indicated that 1200 nm modulation depth is the limit of SRG amplitude possible to achieve in the **azo(HEMA-*co*-MMA)** layer under the applied experimental conditions involving excitation of the n–π* electronic transitions of both trans and cis azo moieties. Inscribing the spatially periodic modulations with a depth up to 1200 nm (by controlling the irradiation time) offers an easy way to fabricate phase diffraction gratings characterized by complete redirection of the probe red light from specific propagation directions: either the zeroth or the first orders into the other remaining ones.

### Supplementary Information

Supplementary Information.

## Data Availability

Sequence data that support the findings of this study have been deposited in the Repository for Open Data (RepOD)—https://doi.org/10.18150/3B7ZZS.
