# Supplementary material for: Photoresponse of new azo pyridine functionalized poly(2-hydroxyethyl methacrylate-co-methyl methacrylate)
Source: Sci Rep. 2024 Apr 20;14:9078. doi: 10.1038/s41598-024-59704-1 (PMC11032328; doi:10.1038/s41598-024-59704-1)
Supplement: Supplementary file 1 — Supplementary Information. [file 41598_2024_59704_MOESM1_ESM.pdf]

Supporting information

for

Photoresponse of new azo pyridine functionalized poly(2-hydroxyethyl methacrylate-*co*-methyl methacrylate)

Jolanta Konieczkowska[1*], Dorota Neugebauer[2], Anna Kozanecka-Szmigiel[3], Aleksy Mazur[2], Sonia Kotowicz[4], Ewa Schab-Balcerzak[1*]

[1]*Centre of Polymer and Carbon Materials, Polish Academy of Sciences, 34 M. Curie-Sklodowska Str., 41-819 Zabrze, Poland*

[2] *Department of Physical Chemistry and Technology of Polymers, Faculty of Chemistry, Silesian University of Technology, ks. Marcina Strzody 9, 44-100 Gliwice, Poland*

[3] *Faculty of Physics, Warsaw University of Technology, 75 Koszykowa Str., 00-662 Warsaw, Poland*

[4] *Institute of Chemistry, University of Silesia, 9 Szkolna Str., 40-006 Katowice, Poland*

Figure and Table of Contents

Fig. S1. X-ray diffraction pattern for **azo(HEMA-*co*-MMA)**.

Fig. S2. UV-Vis spectra of **azo(HEMA-*co*-MMA)** in NMP solution and in film after 6 minutes of UV-light irradiation.

1. Experimental section

1.1. Materials

Methyl methacrylate (MMA) and 2-(hydroxyethyl) methacrylate (HEMA) (both from Alfa Aesar) were desiccated using 4Å molecular sieves (Chempur). Copper(I) bromide, dinonylo-2,2′-bipyridine (dNbpy), ethyl 2-bromoisobutyrate (EBiB), anisole triphenylphosphine (PPh_3), diethyl azodicarboxylate (DEAD), anhydrous N-methyl-2-pyrrolidinone (NMP), tetrahudrofuran (THF) were received from Sigma-Aldrich. 4-(4-hydroxyphenylazo)pyridine (denoted as **AzPy**) was synthesized and characterized in our previous work [1].

1.2. Synthesis of p(HEMA-*co*-MMA)

Comonomers HEMA (5.88 mL, 48.35 mmol) and MMA (5.15 mL, 48.35 mmol), anisole (1.1 mL), dNbpy (131.71 mg, 32×10^{-2} mmol) and CuBr (23.12 mg, 16×10^{-2} mmol) were placed

into a Schlenk flask and degassed by two freeze–pump–thaw cycles. Next, the EBiB initiator (23.97 µL, 16×10^{-2} mmol) was introduced into the mixture to start polymerization at 70 °C in an oil bath. The reaction was stopped after 2 hours by exposure to air. The reaction mixture diluted in THF was passed through a neutral alumina column to remove the copper catalyst, then the polymer was precipitated in diethyl ether and vacuum dried.

^{1}H NMR of p(HEMA-*co*-MMA) (DMSO-d_6, δ, ppm): 4.87 (1H, -CH_2-O**H**), 3.91 (2H, -C**H$_2$**-OH), 4.08 (2H, -COO-C**H**$_2$-), 3.52 (3H, -O-C**H$_3$**), 1.94-1.57 (2H, -C**H$_2$**- in backbone), 1.4-0.51 (3H, -C**H$_3$** in backbone). FT-IR (cm^{-1}): 3600-3100 ν(O-H); 3000-2800 ν(C-H); 1750 ν(C=O); 1150 ν(C-O). GPC-MALLS(THF): M_n= 28 000 g/mol, M_w/M_n=1. GPC(THF): M_n= 16 900 g/mol, M_w/M_n= 1.41. standard: polystyrene standards (580 – 300,000 g/mol).

	conversion (%)			DP		F_{HEMA}	$M_{n,NMR}$ (g/mol)
	HEMA	MMA	total	HEMA	MMA		
P(HEMA-*co*-MMA)	42	45	43.5	126	134	0.48	30000

Conditions: $[HEMA+MMA]_0:[EBiB]_0:[CuBr]_0:[dNbpy]_0$ = 300+300:1:1:2, [HEMA+MMA]:anisol= 10:1 v/v, temperature 70 °C

1.3. Synthesis of azo-HEMA-*co*-MMA

Azo-HEMA-*co*-MMA synthesized *via* the Mitsunobu reaction as was described earlier [2]. The **p(HEMA-*co*-MMA)** precursor (1.39 mmol; 0.3 g); 4-(4-hydroxyphenylazo)pyridine (1.4 mmol; 0.279 g); and PPh_3 (2.8 mmol; 0.624 g) were dissolved in NMP at 85 °C under reflux and in a nitrogen atmosphere. Next, DEAD (2.8 mmol; 0.48 ml) was added dropwise to the mixture, and the Mitsunobu reaction was carried out at 85 °C for 48 h. The **azo-HEMA-*co*-MMA** was precipitated in methanol and purified in a Soxhlet's apparatus for a few days. Products were dried in an oven at 50 °C for 24 h. Yield: 45%.

azo-HEMA-*co*-MMA: ^{1}H NMR (600 MHz, DMSO-d_6), *δ* [ppm]: 0.71-1.35 (3H, C**H$_3$** in backbone), 1.50-2.01 (4H, C**H$_2$** in backbone); 3.34-3.42 (3H, -O-C**H$_3$**), 4.07-4.22 (4H, -COO-C**H$_2$**/ -C**H$_2$**-O-AzPy), 7.00 (2H, ArH), 7.53 (2H, ArH), 7.76 (2H, ArH), 8.67 (2H, ArH). FT-IR (cm^{-1}): 2993, 2886 ν (C-H); 1729 ν(C=O); 1145 ν(C-O); 1587 ν(N=N); 988 ν(Py).

1.4. Polymer film preparation

The homogenous solutions of polymers in NMP were filtered through 0.45 mm membranes and cast onto clean glass substrates (2×2cm). The residual solvent was removed from films by heating them for 24 h at 85 °C in vaccum, near the polymer T_g, that is the temperature AT which the polymer segmental motion is large enough to free possibly trapped solvent molecules.
In the surface relief grating recording experiment, the thickness of the prepared polymer layer was 2.7 μm. It was determined using a Dektak XT stylus profiler (by scanning the surface of the polymer layer with an intentionally made scratch).

2. Measurements

2.1 Polymer characterization

^{1}H NMR spectroscopy experiments of the investigated materials were carried out on an Avance II UltraShield Q3 Plus Bruker MT 600 MHz spectrometer (Germany). Tetramethylsilane (TMS) and deuterated dimethyl sulfoxide (DMSO-d_6) were used as an internal standard and as a solvent, respectively.
FTIR-ATR spectroscopy data were received by using a Nicolet 6700 FTIR apparatus (Thermo Scientific, MA, USA). The FTIR spectra were acquired in the range of 4000 – 500 cm^{-1}, at the resolution of 0.09 cm^{-1} for 64 accumulated scans.
Molecular weights (Mn, SEC) and dispersity indices (Đ) were determined by size-exclusion chromatograph (SEC, Ultimate 3000) equipped with an isocratic pump, autosampler, degasser, thermostatic box for columns and differential refractometer RefractoMax 521 Detector and DAWN®, Multi-Angle Laser Light Scattering (MALLS) detector. ASTRA data analysis software was used for data collecting and processing. The RID calculated molecular weight was based on calibration using linear polystyrene standards (Mp = 580 – 3000 000 g/mol). Pre-column guard 5μm 50 × 7.5 mm and double PLGel 5μm MIXED-C and MIXED-D 300 × 7.5 mm column were used for separation. The measurements were carried out in THF (HPLC grade) as the solvent at 35 °C with flow rate of 1 mL/min.
Wide-angle X-ray diffraction (WAXD) analysis was performed using an HZG-4 diffractometer (Carl Zeiss, Jena, Germany). The samples were examined working on Bragg geometry of 2θ from 5 to 50° using Ni-filtered Cu-Kα radiation with a wavelength λ = 1.54051 Å.
Differential Scanning Calorimetry (DSC) data were recorded on a TA-DSC 2010 apparatus (TA Instruments, Newcastle, DE, USA). Before the analysis, the polymer samples were vacuum dried at 60 °C for 24 h. The samples were analyzed with a heating rate of 20 °C min^{-1}. The glass transition temperature (T_g) was taken as a midpoint of the heat capacity step change registered in a second run.

Thermogravimetric analysis (TGA) was done with a TA TGA55 apparatus with a heating rate of 15 °C·min^{-1} in a constant stream of nitrogen (25 ml·min^{-1}) and a temperature range from 25 °C to 800 °C. The compounds before measurement were dried in an oven at 100 °C or 80 °C for 24 h in a glass vessel.

UV-Vis spectroscopy. The measurements of UV–Vis absorption spectra were measured for polymer solutions in NMP (c=10^{-5} mol/l) and films casting on a glass substrate using a Jasco V-750 (Jasco Inc.). The cell path used for the spectroscopic measurement of the solution was 10 mm.

Atomic Force Microscopy. The thickness of the film was determined by atomic force microscopy (AFM) using a TopoMetrix Explorer device, operating in contact mode, in air, and constant force regime.

2.2. Loading level of 4-(4-hydroxyphenylazo)pyridine

The chromophore loading level of the azo polyimides was determined by the UV–Vis spectroscopy using the Lambert–Beer Law [6]. The degree of the chromophore substitution in the polymer was calculated from the calibration curves obtained from the UV–Vis spectra of the chromophore solution with various concentrations (1×10^{-6} – 1×10^{-4} mol/l) for the 4-(4-hydroxyphenylazo)pyridine (equation of the calibration curve was y = 9.2756x – 0.449). From the comparison of the peak absorbance of the polymer in solution with the known concentration and the calibration curve, the degree of the functionalization was estimated. The absorbance of azo polymer (c = 10^{-5} mol/l) was 0.1663 for **azo(HEMA-*co*-MMA).**

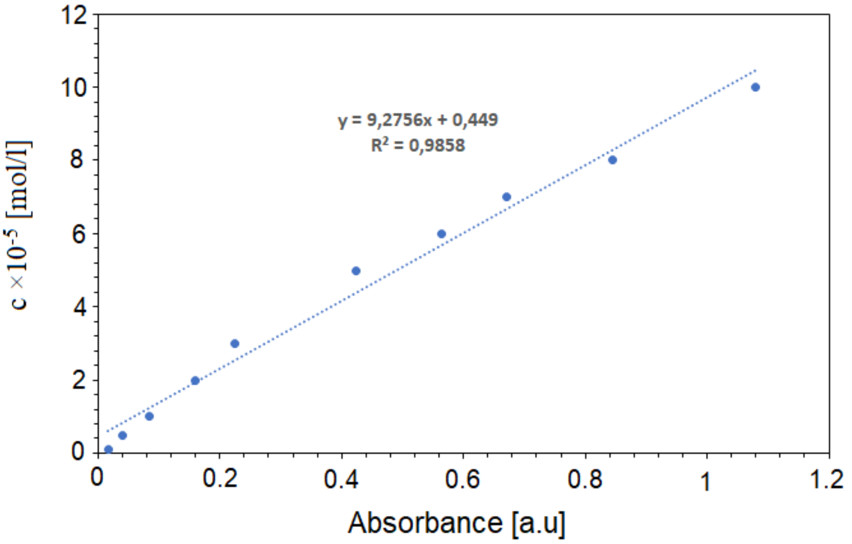

Fig. S1. Calibration plot for 4-(4-hydroxyphenylazo)pyridine in NMP.

2.3. *Cis-trans* isomerization measurements

The measurements of UV–Vis absorption spectra were measured for polymer films cast on a glass substrate placed in the darkroom for at least 24 hours. The thickness of the film was ca. 300 nm (Fig. S5). The *trans-cis* and *cis-trans* reactions were monitored at room temperature (25 °C). The measurement procedure was as follows: in the first step, the absorption spectra of the polymer films were recorded without any irradiation. Then the *cis*-isomers were generated by 365 nm (P = 2.9 W) until reaching the photostationary state. The distance between sample and diode was 7 cm. The photostationary state was achieved after 6 min of diode LED irradiation. Increasing the exposure time did not increase the number of *cis*-isomers in the sample compared to the 6-minute exposure time. Then *cis-trans* dark-return isomerization at room temperature was monitored by recording changes in the absorption spectrum for 48h after turning off the excitation light.

By monitoring the changes in the absorbance at the wavelength corresponding to the absorption maximum of the *trans*-form before and immediately after light irradiation, the conversion efficiency of the *trans*-form to the *cis*-form was determined according to the following equation:

$$P = \frac{A_0 - A_t}{A_0} \times 100\%$$

where A_0 and A_t correspond to the normalized absorbance before and after 6 min irradiation, respectively.

2.4. Surface relief grating (SRG) inscription

The formation of SRGs in the 2.7 µm-thick polymer layer cast onto a glass substrate was studied in an experimental set-up presented recently [3]. An ultra-low noise narrow linewidth 457 nm DPSS laser (CNI) with a circular spot was used as the excitation light source. The DPSS 457 nm laser with a circular spot was used as the excitation light source. After splitting the laser beam into two of equal intensity, metallic mirrors directed the beams to the sample surface. Two properly aligned quarter wave plates inserted in each beam path set the beams' polarization as opposite circular. In the arranged recording geometry, the interfering beams crossed at a small angle of 2.7^o, forming an almost purely linearly polarized optical field characterized by a continuously rotating polarization azimuth [4] and a 9.7 µm spatial periodicity (according to the formula: $\Lambda=\lambda/(2\sin(\theta/2))$, where λ is the recording wavelength and

θ is the crossing angle [5]). The diameter ($1/e^2$) of each interfering beam at the sample plane was 2.8 mm, and their intensity was 100 mW/cm^2. During over 4-hour exposure, another laser beam of 690 nm wavelength and horizontal polarization was incident at the centre of the interference region. The beam was focused to the size of ca. 110 μm to monitor the SRG build-up process locally. The optical power of the red beam diffracted from the grating into 0th, $+1^{st}$ and -2nd orders was measured as a function of time using three silicon photodetectors. The surface morphology was examined after irradiation with a Dektak XT stylus profiler.

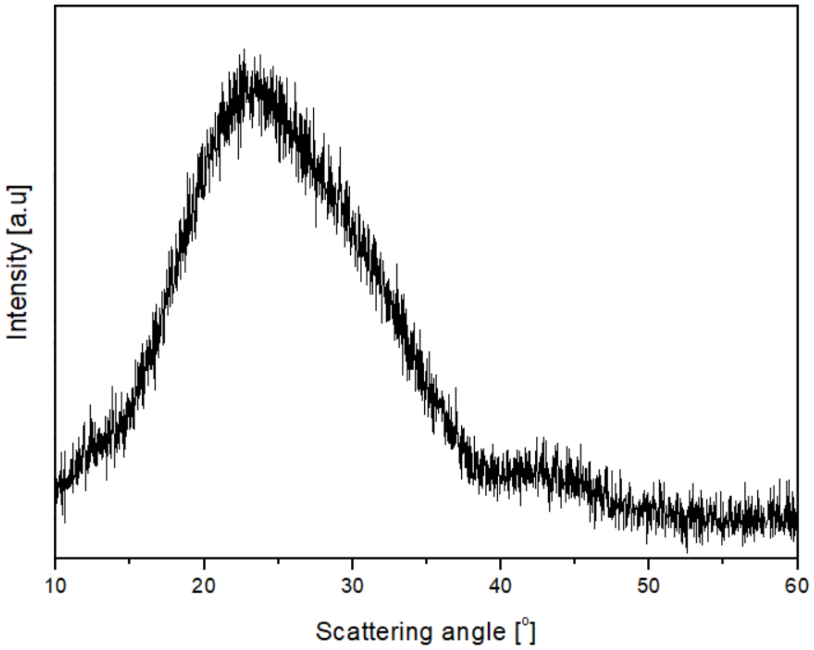

Fig. S2. X-ray diffraction pattern for **azo(HEMA-*co*-MMA)**.

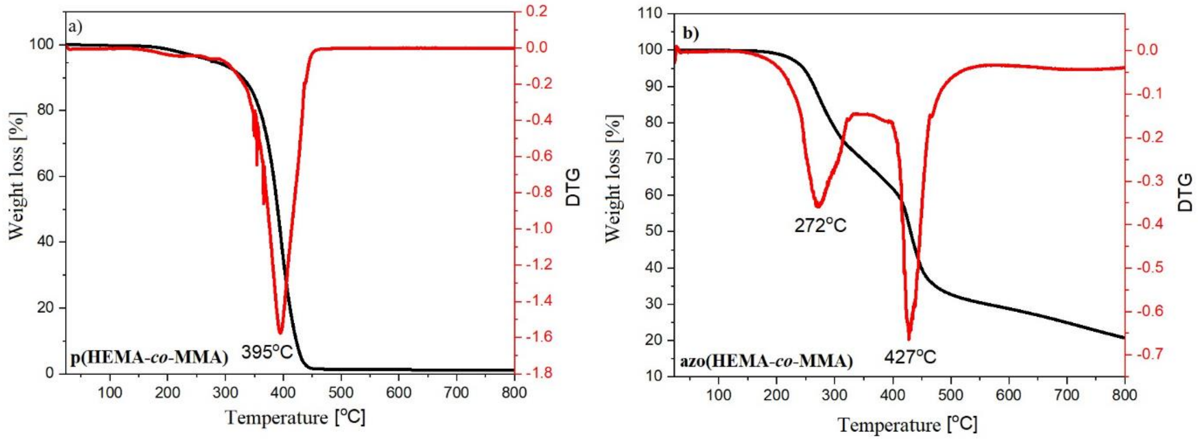

Fig. S3. Thermogravimetric curves for (a) **p(HEMA-*co*-MMA)** and (b) **azo(HEMA-*co*-MMA)**

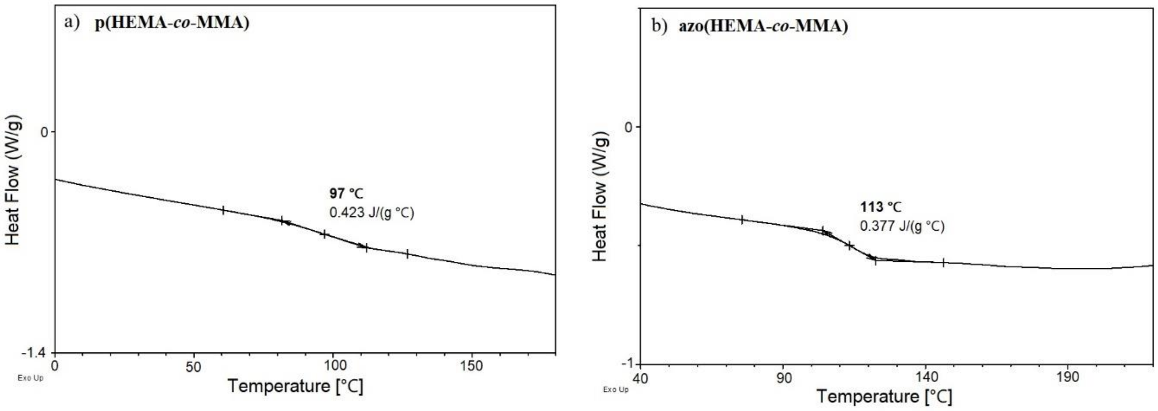

Fig. S4. DSC for (a) **p(HEMA-*co*-MMA)** and (b) **azo(HEMA-*co*-MMA)**. Heating rate of 20 °C min^{-1}.

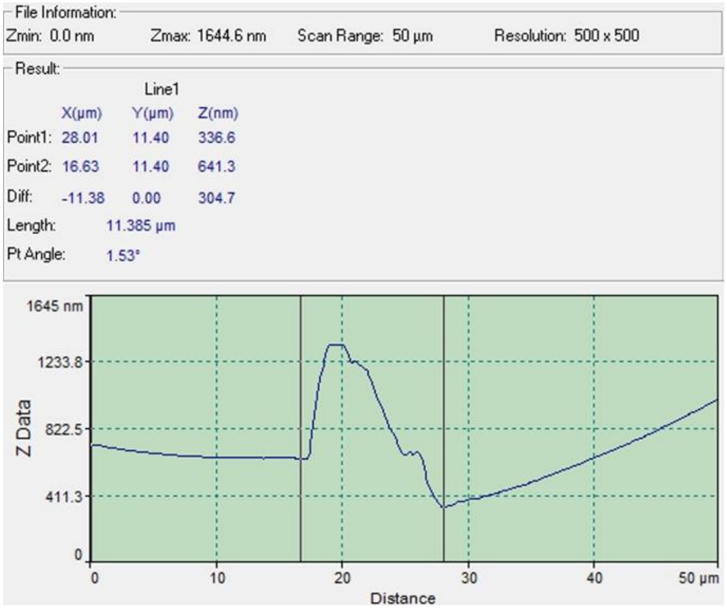

Fig. S5. Thickness of the **azo(HEMA-*co*-MMA)** film used in optical measurements.

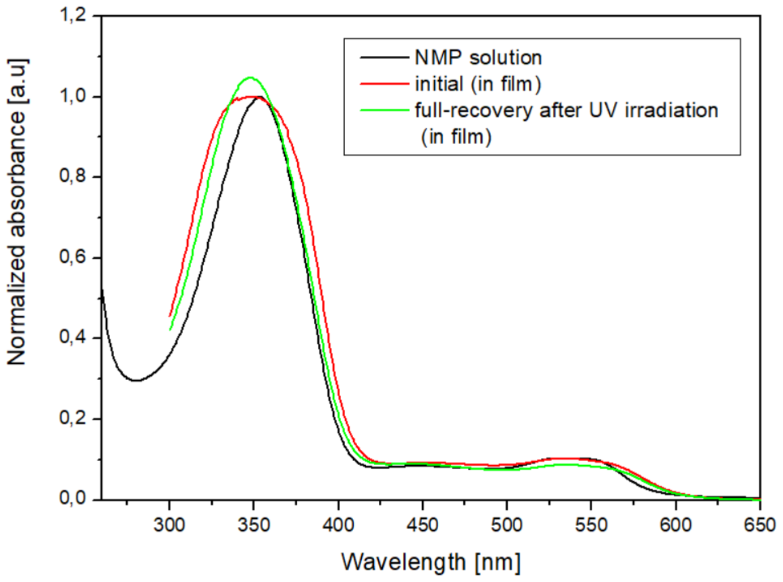

Fig. S6. UV-Vis spectra of **azo(HEMA-*co*-MMA)** in NMP solution (c = 10^{-5}) , initial (in film) and after full-recovery to the trans-isomer (in film).

[1] E. Schab-Balcerzak, J. Konieczkowska, M. Siwy, A. Sobolewska, M. Wojtowicz, M. Wiacek, Opt. Mater. 36 (2014) 892.

[2] K. Nocoń-Szmajda, A. Jankowski, A. Wolińska-Grabczyk, J. Konieczkowska, Guest-host and functionalized side-chain azopolyimide membranes for controlled gas separation, Polymer 229 (2021) 124012.

[3] A. Kozanecka-Szmigiel, A. Hernik, K. Rutkowska, J. Konieczkowska, E. Schab-Balcerzak, D. Szmigiel, Surface relief modulated grating in azo polymer - from the tailoring of diffraction order to reshaping of a laser beam, Materials 15 (2022) 1.

[4] M. Xu, D. K. de Boer, C. M. van Heesch, A. J. Wachters and H. P. Urbach, Photoanisotropic polarization gratings beyond the small recording angle regime, Opt. Express, 18 (2010) 6703.

[5] Viswanathan, N.K. Balasubramanian, S. Li, L. Tripathy, S.K. Kumar, J. A detailed investigation of the polarization-dependent surface-relief-grating formation process on azo polymer films. Japanese Journal of Applied Physics 38 (1999) 5928.

[6] [18] E. Schab-Balcerzak, M. Siwy, M. Kawalec, A. Sobolewska, A. Chamera, A. Miniewicz, Synthesis, Characterization, and Study of Photoinduced Optical Anisotropy in Polyimides Containing Side Azobenzene Units, J. Phys. Chem. A 113 (2009) 8765.
